# Red Cell Distribution Width is Associated with Bleeding Complications after Coronary Artery Bypass Grafting

**DOI:** 10.1093/icvts/ivaf299

**Published:** 2025-12-22

**Authors:** Alexandra Aldis Heimisdottir, Luis Gisli Rabelo, Matthildur Maria Magnusdottir, Anders Jeppsson, Tomas Gudbjartsson

**Affiliations:** Department of Cardiothoracic Surgery, Landspitali University Hospital, Reykjavik, 101, Iceland; Faculty of Medicine, University of Iceland, Reykjavik, 101, Iceland; Department of Cardiothoracic Surgery, Landspitali University Hospital, Reykjavik, 101, Iceland; Faculty of Medicine, University of Iceland, Reykjavik, 101, Iceland; Faculty of Medicine, University of Iceland, Reykjavik, 101, Iceland; Department of Cardiothoracic Surgery, Sahlgrenska University Hospital, Gothenburg, 413 45, Sweden; Department of Molecular and Clinical Medicine, Institute of Medicine, Sahlgrenska Academy, University of Gothenburg, Gothenburg, 413 45, Sweden; Faculty of Medicine, University of Iceland, Reykjavik, 101, Iceland

**Keywords:** CABG, RDW, bleeding, transfusion, re-exploration, perioperative morbidity

## Abstract

**Objectives:**

Elevated red cell distribution width (RDW) has been associated with adverse outcomes in coronary artery disease but its role in bleeding after cardiac surgery is unclear. We evaluated whether preoperative RDW predicts bleeding after isolated coronary artery bypass grafting (CABG).

**Methods:**

This was a nationwide retrospective study of patients undergoing isolated CABG in Iceland, 2003-2019. RDW was analysed continuously (per 1% increase) and dichotomized (>14.0% vs ≤14.0%). Primary bleeding outcomes included transfusion >4 red blood cell units, re-exploration for bleeding, and chest tube output >1000 mL/24h. Multivariable logistic regression adjusted for demographics, comorbidities, operative urgency, cardiopulmonary bypass, and perioperative factors.

**Results:**

The study included 1929 patients. Elevated RDW was associated with older age, anaemia, comorbidities, and urgent procedures. After adjustment, higher RDW predicted transfusion >4 RBC units (OR 1.25 per 1%, OR 1.72 for >14.0%), re-exploration (OR 1.30 per 1%, OR 2.39 for >14.0%), and chest tube output >1000 mL/24 h (OR 1.13 per 1%, OR 1.34 for >14.0%). RDW was also associated with greater platelet/plasma use, longer ICU stay, and major complications, but not to 30-day mortality (OR 1.21, 95% CI 0.55-2.52).

**Conclusions:**

Elevated RDW was independently associated with multiple bleeding complications after CABG. RDW may serve as a simple, inexpensive biomarker to improve preoperative bleeding risk stratification in CABG patients.

## Graphical abstract

**Figure:**
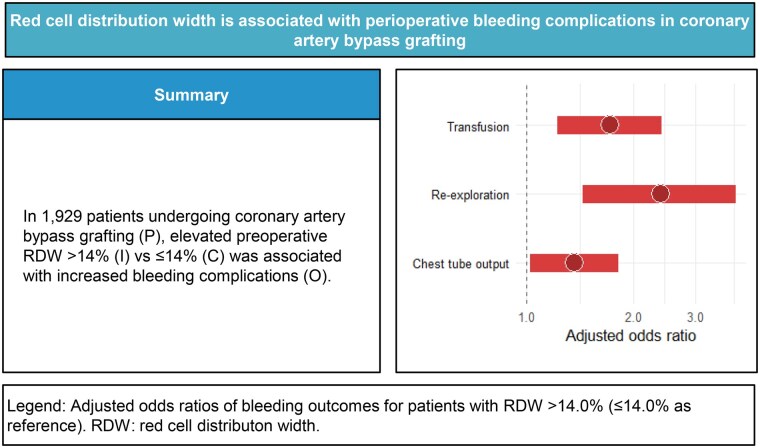

## INTRODUCTION

Coronary artery bypass grafting (CABG) is the recommended treatment for selected patients with advanced coronary artery disease (CAD) and is performed globally with generally favourable outcomes.^1^ However, excessive bleeding is common and may require transfusion or re-exploration.^2^^,^^3^ Such bleeding is associated with adverse outcomes, including atrial fibrillation, stroke, myocardial injury, and increased short-term mortality.^4^^,^^5^ Known risk factors include recent antiplatelet use (eg, aspirin, clopidogrel, ticagrelor), renal dysfunction, lower BMI, urgent surgery, prolonged cardiopulmonary bypass (CPB) time, and older age.^4^^,^^6^^,^^7^

Red cell distribution width (RDW), a routinely measured parameter in the complete blood count, reflects variability in red blood cell (RBC) size, and, when elevated, may signify deviations from normal erythropoiesis, inflammation, and oxidative stress.^8–11^ It has been associated with worse outcomes in conditions such as heart failure, myocardial infarction (MI), and CAD, including in patients undergoing percutaneous coronary intervention.^12^^,^^13^ In cardiac surgery, there has been a growing interest in RDW as a possible predictor of postoperative outcomes and prior work has mainly linked RDW to mortality.^10^^,^^14^^,^^15^ A recent systematic review of over 40 000 cardiac surgery patients showed elevated RDW predicted mortality and morbidity, but few studies examined bleeding specifically.^10^ Thus, evidence on perioperative bleeding risk in CABG remains sparse and inconsistent.

Given the clinical relevance of bleeding and the need for improved preoperative risk stratification, identifying novel markers is important. We therefore examined whether preoperative RDW is associated with 3 distinct bleeding outcomes—transfusion >4 RBC units, re-exploration, and chest tube output >1000 mL/24 h—in a large nationwide cohort. We hypothesized that elevated RDW independently predicts bleeding complications and perioperative morbidity.

## METHODS

### Data sources

All patients undergoing isolated CABG at Landspitali University Hospital, Iceland’s sole cardiac surgery centre, between January 1, 2003, and December 31, 2019 were identified from institutional registries and a national cardiac surgery database. Demographics, comorbidities, laboratory values, medications, operative details, and outcomes were collected retrospectively from electronic medical records across all Icelandic hospitals. Eight primary surgeons performed all cases; outcomes did not differ meaningfully between them, and mixed-effects models confirmed minimal surgeon-level variability.

The study was approved by the Icelandic National Bioethics Committee (VSN10-009, March 11, 2025) and the Landspitali Bioethics Committee, with informed consent waived due to its retrospective design. Data were obtained from existing registries and handled in line with the Declaration of Helsinki. No new biobank was created; use of stored data complied with the WMA Declaration of Taipei under ethics committee oversight. Reporting adheres to the Strengthening the Reporting of Observational Studies in Epidemiology (STROBE) statement.^16^

### Study population

Eligible patients were ≥18 years undergoing primary, isolated CABG, including off-pump CABG (OPCAB). Baseline characteristics included age, sex, BMI, comorbidities, cardiac history, and symptom severity (Canadian Cardiovascular Society and the New York Heart Association Functional classifications).^17^^,^^18^ Laboratory values included preoperative haemoglobin, creatinine and, from 2005, platelet count (×10^9^/L). Estimated glomerular filtration rate (eGFR) was calculated by Chronic Kidney Disease Epidemiology Collaboration (CKD-EPI); impaired renal function was defined as eGFR <60 mL/min/1.73 m^2^.^19^ CAD severity and left ventricular ejection fraction (LVEF) were obtained from angiography and echocardiographic reports. Medication data included recent antiplatelet and anticoagulant use; ticagrelor and prasugrel were not available in Iceland during the study period. EuroSCORE II was calculated for all patients.^20^ Operative data included CPB time/status, number of distal anastomoses, and left internal mammary artery use.

### Exposure of interest

The primary exposure was preoperative RDW measured within 30 days of surgery. RDW was analysed both continuously (per 1% increase) and dichotomized (>14.0% vs ≤14.0%), with 14.0% selected by ROC/Youden index and consistent with prior literature.^3^^,^^14^^,^^15^ Model fit was compared across categorizations, also with RDW categorized as quartiles, using Akaike (AIC) and Bayesian (BIC) information criteria, which showed little difference between models, although continuous models provided the best fit by AIC and BIC. The binary cut-off was retained for clinical interpretability and comparability with prior studies.^3^^,^^14^ Patients missing preoperative RDW, bleeding outcomes, or with extreme chest tube output (>5L) were excluded (*n* = 43).

### Outcome measures

Primary bleeding outcomes were: (1) transfusion of >4 units of RBC within 7 days; (2) re-exploration for bleeding; and (3) chest tube output >1000 mL/24 h. Institutional transfusion thresholds were haemoglobin <90 g/L (or <100 g/L with active major bleeding) until 2010, later revised to <80 g/L (or <100 g/L with active major bleeding). The >4 units definition aligns with the Universal Definition of Perioperative Bleeding in Cardiac Surgery.^21^ RBC transfusion counts were also modelled using quasi-Poisson regression.

Secondary outcomes included platelet or plasma transfusion ≥2 units, 30-day mortality, ICU and hospital length of stay, and complications. Prolonged LOS was defined as ≥75th percentile (>1 day in ICU, >11 days in hospital). Acute kidney injury (AKI) followed the Kidney Disease Improving Global Outcomes (KDIGO) criteria.^22^ Minor complications included new-onset atrial fibrillation and flutter, superficial sternal wound or harvest site infections, or pneumonia; major complications included sternal dehiscence, deep sternal wound infection, perioperative stroke (neurological signs that persisted for more than 24 h), MI, AKI requiring dialysis, or multiorgan failure. MI was defined per the Fourth Universal Definition.^23^

### Statistical analyses

Statistical analyses were performed in R, version 4.4.2, with significance set at *P* < .05. Categorical variables were described as counts/percentages and compared by *χ*^2^ or Fisher’s exact tests. Continuous variables were described as means ± standard deviations or medians with interquartile ranges (IQRs) and compared with *t*-tests, analysis of variance tests, or Kruskal-Wallis as appropriate. Missing data were imputed using the random forest method.^24^

Correlation between RDW and haemoglobin was assessed by Pearson coefficients and linear regression with restricted cubic splines. Receiver operating characteristic (ROC) curve analysis evaluated the discriminative ability of RDW to identify WHO-defined anaemia.^25^ The area under the curve (AUC) was calculated and the optimal RDW cut-off for anaemia detection was determined using ROC analysis with Youden’s index.

Multivariable logistic regression estimated odds ratios (ORs) and 95% confidence intervals (CIs) for bleeding and other complications. Covariates were chosen for clinical relevance and literature precedence: age, sex, BMI, year of surgery, preoperative anaemia, LVEF, eGFR, operative urgency, CPB time/status, diabetes, COPD, EuroSCORE II, and recent use of aspirin, clopidogrel, heparin, or warfarin. Sensitivity analyses included: (i) restriction to 2005-2019 with adjustment for platelet count, (ii) stratification by era (2003-2010 vs 2011-2019), and (iii) subgroup analysis of on-pump vs OPCAB patients. In the on-pump subgroup analysis, OPCAB vs on-pump was included in all analyses; in the on-pump subgroup, CPB time replaced CPB status. Collinearity was checked with variance inflation factors (all <2.5). Model discrimination was assessed using *c*-statistics (AUC) and model calibration was assessed using LOESS curves for all three primary models.

Thirty-day mortality was analysed separately using logistic regression, adjusting for age, sex, EuroSCORE II, and impaired renal function. A limited covariate set was used, due to the relatively small number of events. The AUC was calculated, along with a 95% CI.

## RESULTS

### Baseline characteristics

The final cohort consisted of 1929 patients; 387 (20.1%) had elevated RDW (>14.0%). Median age was 67 years and 82.6% were male. As shown in **Table 1**, elevated RDW was associated with older age, female sex, anaemia, COPD, diabetes, renal dysfunction, and urgent/emergent surgery. These patients were more often on preoperative antiplatelet therapy, had longer CPB times and higher EuroSCORE II. OPCAB accounted for 17.4% (*n* = 336) of the surgeries. Missing values are detailed in **Table S1**.

**Table 1.** Preoperative and Operative Characteristics by Preoperative RDW

	Overall (*n* = 1929)	Normal RDW (*n* = 1542)	Elevated RDW (*n* = 347)	*P*-value
Preoperative				
RDW (%)	13.2 ± 1.3	12.8 ± 0.8	15.0 ± 1.2	**<.001**
Female	336 (17.4)	233 (15.1)	103 (26.6)	**<.001**
Age (years)	67 [60-73]	66 [60-72]	72 [64-76]	**<.001**
BMI (kg/m^2^)	27.8 [25.4-30.9]	27.8 [25.5-30.9]	27.9 [25.1-31.1]	.357
Preoperative haemoglobin (g/L)	140.9 ± 14.6	142.9 ± 13.2	133.3 ± 16.7	**<.001**
Anaemia	377 (19.5)	244 (15.8)	133 (34.4)	**<.001**
Preoperative platelet count (×10^9^/L)	231 ± 66	230 ± 63	234 ± 78	.315
LVEF				**.029**
>50%	1203 (62.4)	975 (65.1)	228 (61.0)	
30%-50%	567 (29.4)	451 (30.1)	116 (31.0)	
≤30%	102 (5.3)	72 (4.8)	30 (8.0)	
NYHA score III/IV	1128 (58.5)	902 (58.5)	226 (58.4)	.999
CCS score III/IV	1355 (70.2)	1067 (69.2)	288 (74.4)	.052
EuroSCORE II (%)	1.4 [0.9-2.5]	1.3 [0.9-2.2]	2.0 [1.2-4.1]	**<.001**
Three-vessel disease and/or left main stenosis	1716 (89.0)	1377 (89.3)	339 (87.6)	.387
Previous myocardial infarction	415 (21.5)	320 (20.8)	95 (24.5)	.120
Current smokers	436 (22.6)	333 (22.0)	103 (27.2)	**.042**
COPD	145 (7.5)	101 (6.6)	44 (11.5)	**.002**
Cardiac valve disease^a^	72 (3.7)	53 (3.4)	19 (4.9)	.225
Hypertension	1270 (65.8)	1011 (52.4)	259 (65.3)	.588
Diabetes mellitus	359 (18.6)	271 (17.7)	88 (23.1)	**.018**
Impaired renal function^b^	365 (18.9)	234 (15.2)	131 (33.9)	**<.001**
Preoperative use of aspirin	988 (51.2)	738 (50.5)	250 (66.0)	**<.001**
Preoperative use of clopidogrel	88 (4.6)	61 (4.2)	27 (7.1)	**.024**
Preoperative use of anticoagulants^c^	358 (18.6)	307 (21.0)	51 (13.5)	**.001**
Operative				
Operative status				**<.001**
Elective	851 (44.1)	710 (46.0)	151 (36.4)	
Urgent	972 (50.4)	762 (49.4)	210 (54.3)	
Emergent	106 (5.5)	70 (4.5)	36 (9.3)	
No. of distal anastomoses, median [range]	3 [3-4]	3 [3-4]	4 [3-4]	.676
Mammary artery used	1815 (94.1)	1454 (94.4)	361 (93.3)	.495
CPB time (min)	87 [72-109]	86 [72-106]	92 [75-117]	**<.001**
Cross-clamp time (min)	46 [37-58]	46 [37-58]	49 [39-61]	**<.001**

Mean ± standard deviation, median [IQR] or number (%), unless otherwise specified. Bold values indicate statistically significant results (*P* < .05).

a Mild to moderate aortic stenosis or mitral regurgitation, not hemodynamically significant or requiring surgery.

b eGFR <60 mL/min/1.73 m^2^.

c Warfarin, heparin, or low-molecular-weight heparin.

Abbreviations: BMI = body mass index; CCS = Canadian Cardiovascular Society; COPD = chronic obstructive pulmonary disease; CPB = cardiopulmonary bypass; eGFR = estimated glomerular filtration rate; EuroSCORE = European System for Cardiac Operative Risk Evaluation; IQR = interquartile range; LVEF = left ventricular ejection fraction; NYHA = New York Heart Association; RDW = red cell distribution width.

Red cell distribution width correlated negatively with haemoglobin (*r* = −0.30, *P* < .001) and this association persisted when restricting the analysis to haemoglobin values <150 g/L (*r* = −0.31, *P* < .001). ROC analysis showed poor discrimination for anaemia (AUC 0.59, 95% CI 0.556-0.620). Restricted cubic splines indicated RDW decreased with haemoglobin up to ∼150 g/L, then plateaued, as shown in **Figure S1**.

### Bleeding outcomes

**Figure 1.**
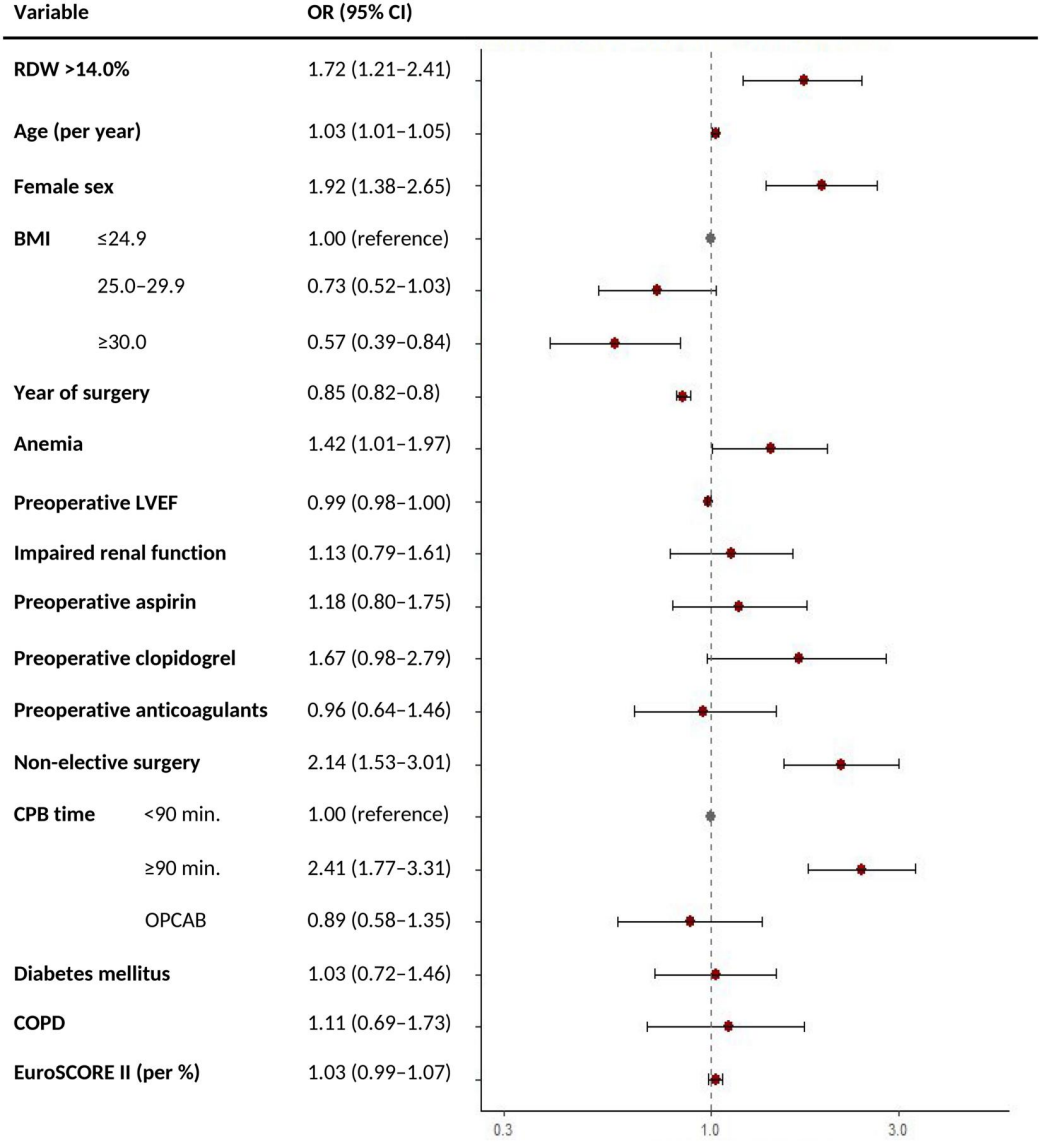
Forest Plot of Adjusted ORs (95% CIs) for Transfusion. Abbreviations: BMI = body mass index; COPD = chronic obstructive pulmonary disease; CPB = cardiopulmonary bypass; EuroSCORE = European System for Cardiac Operative Risk Evaluation; LVEF = left ventricular ejection fraction; OPCAB = off-pump coronary artery bypass; OR = odds ratio; RDW = red cell distribution width

Bleeding outcomes are shown in **Table 2**, with group comparisons in **Table S2**. Overall, 58.1% of patients were transfused; 14.3% received >4 RBC units. After adjustment, each 1% RDW increase raised odds of >4 units transfusion by 25% (OR 1.25, 95% CI: 1.12-1.39) and RDW >14.0% carried an OR of 1.72 (95% CI: 1.22-2.41) compared to RDW ≤14.0%. Adjusted ORs are shown in a forest plot in **Figure 1**. Quasi-Poisson models confirmed higher transfusion counts with elevated RDW (IRR 1.33, *P* < .01).

**Table 2.** Unadjusted and Adjusted Analyses for Bleeding Outcomes by Preoperative RDW

	Continuous RDW (per 1% increase)		Dichotomized RDW (>14.0% vs ≤14.0%)	
	Unadjusted OR (95% CI)	Adjusted^a^ OR (95% CI)	Unadjusted OR (95% CI)	Adjusted^a^ OR (95% CI)
Transfusion >4 units	1.24 (1.14-1.36)	1.25 (1.12-1.39)	1.99 (1.49-2.64)	1.72 (1.22-2.41)
Re-exploration	1.29 (1.13-1.46)	1.30 (1.11-1.51)	2.42 (1.55-3.73)	2.39 (1.44-3.91)
Chest tube output >1000 mL/24 h	0.97 (0.89-1.05)	1.13 (1.02-1.24)	1.00 (0.77-1.29)	1.36 (1.02-1.82)

a Adjusted for preoperative aspirin, clopidogrel within 5 days, heparin and warfarin; age, sex, BMI, year of surgery, preoperative anaemia, preoperative LVEF, preoperative eGFR, non-elective procedures, cross-clamp time, diabetes mellitus, COPD, and EuroSCORE II.

Abbreviations: BMI = body mass index; COPD = chronic obstructive pulmonary disease; eGFR = estimated glomerular filtration rate; EuroSCORE = European System for Cardiac Operative Risk Evaluation; LVEF = left ventricular ejection fraction; OR = odds ratio; RDW = red cell distribution width.

**Figure 2.**
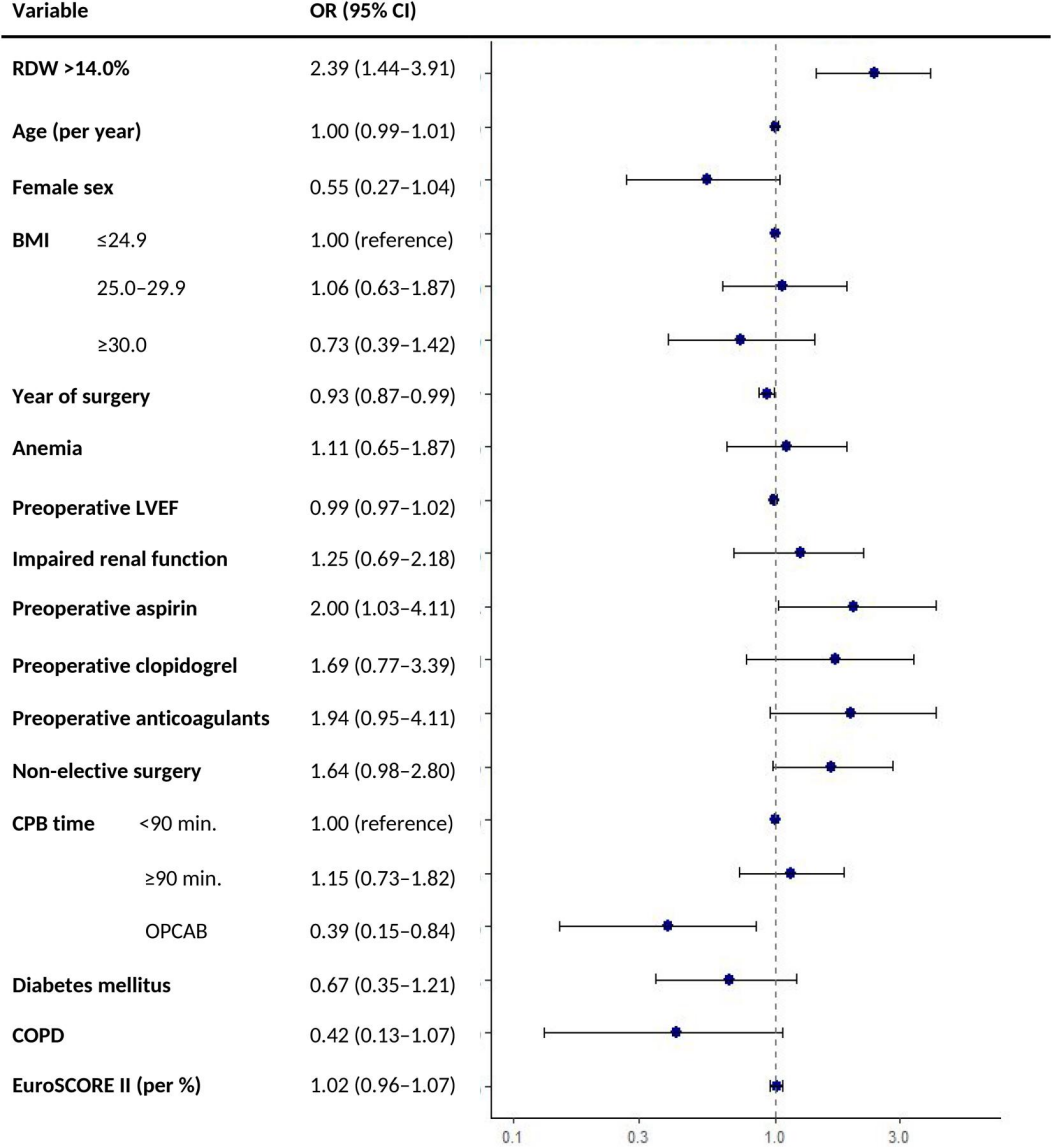
Forest Plot of Adjusted ORs (95% CIs) for Re-exploration. Abbreviations: BMI = body mass index; COPD = chronic obstructive pulmonary disease; CPB = cardiopulmonary bypass; EuroSCORE = European System for Cardiac Operative Risk Evaluation; LVEF = left ventricular ejection fraction; OPCAB = off-pump coronary artery bypass; OR = odds ratio; RDW = red cell distribution width

Re-exploration occurred in 4.8%. Elevated RDW predicted re-exploration, both continuously (OR 1.30, 95% CI: 1.11-1.51 per 1% increase) and dichotomized (OR 2.39, 95% CI: 1.44-3.91). **Figure 2** presents adjusted ORs for re-exploration.

**Figure 3.**
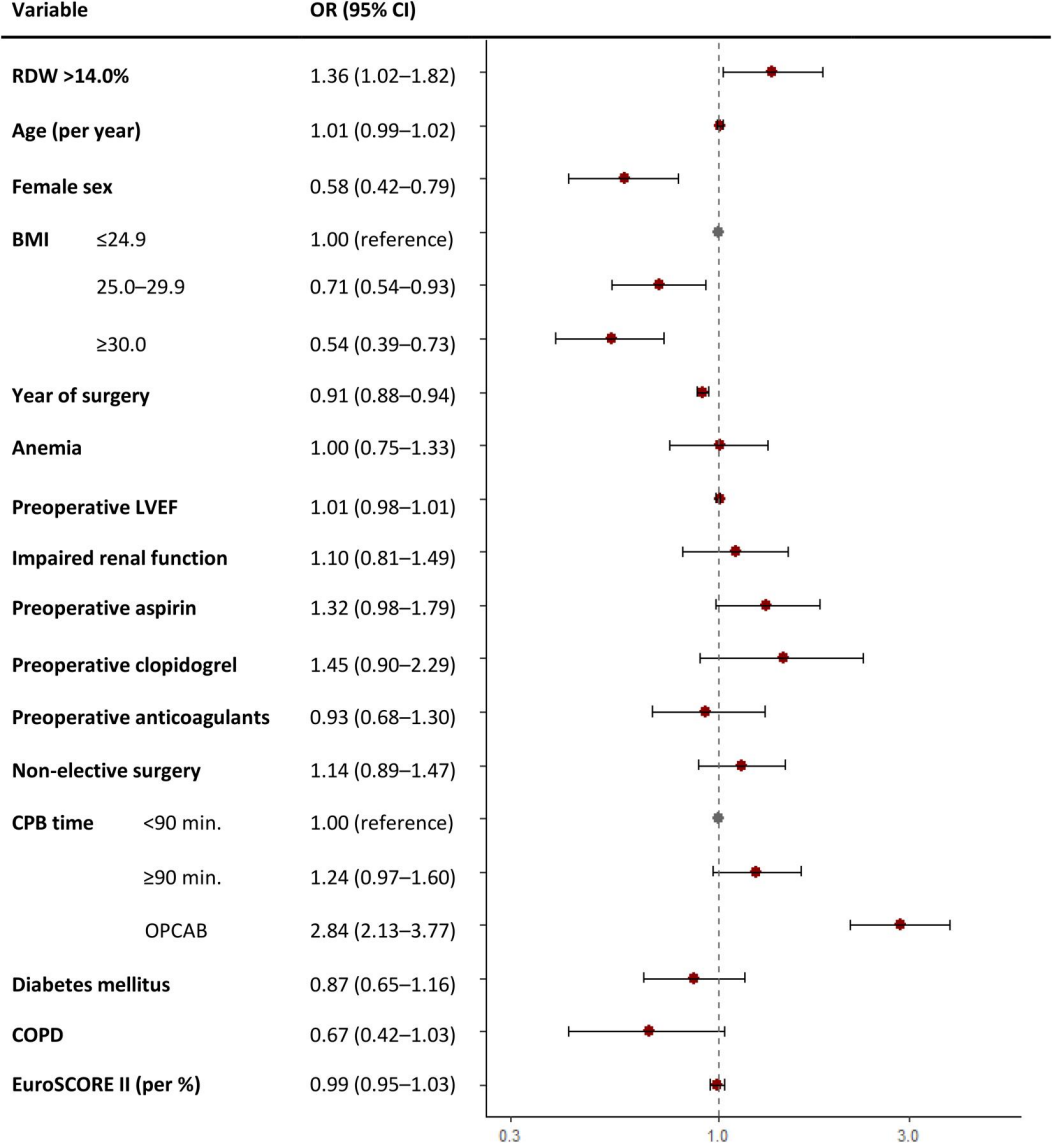
Forest Plot of Adjusted ORs (95% CIs) for Chest Tube Output >1000 ml/24h. Abbreviations: BMI = body mass index; COPD = chronic obstructive pulmonary disease; CPB = cardiopulmonary bypass; EuroSCORE = European System for Cardiac Operative Risk Evaluation; LVEF = left ventricular ejection fraction; OPCAB = off-pump coronary artery bypass; OR = odds ratio; RDW = red cell distribution width

Median chest tube drainage was 730 mL. Elevated RDW was associated with chest tube output >1000 mL/24 h in adjusted analyses (continuous: OR 1.13, 95% CI: 1.02-1.24; dichotomized: OR 1.36, 95% CI: 1.02-1.82). **Figure 3** presents adjusted ORs for postoperative chest tube output.

Model *c*-statistics were 0.677 (chest tube), 0.737 (re-exploration), and 0.763 (transfusion), indicating moderate discrimination. Other bleeding risk factors included anaemia, non-elective status, and CPB ≥90 min. Higher BMI was protective for transfusion; OPCAB reduced re-exploration risk. Female sex was associated with higher odds of transfusion but lower odds of chest tube output >1 L. Calibration was acceptable for 2 models but poorer for re-exploration. See **Figures S2-S4.** 

### Sensitivity and subgroup analyses

Associations between elevated RDW and bleeding outcomes were generally consistent across both eras of 2003-2010 (*n* = 1141) and 2011-2019 (*n* = 815), although specific end-points varied, as detailed in **Table S3**.

In on-pump patients (*n* = 1,593), elevated RDW remained significantly associated with all bleeding outcomes, as presented in **Table S4**. Within the on-pump subgroup, CPB time ≥90 min was additionally linked to transfusion and increased chest tube output. In OPCAB (*n* = 336), event counts were low, and associations were nonsignificant though directionally similar. Interaction testing showed no significant modification by surgical technique.

Including platelet count in a sensitivity analysis from 2005 to 2019 (*n* = 1666), shown in **Table S6**, did not alter results: RDW remained predictive of transfusion and re-exploration.

### Thirty-day mortality

**Figure 4.**
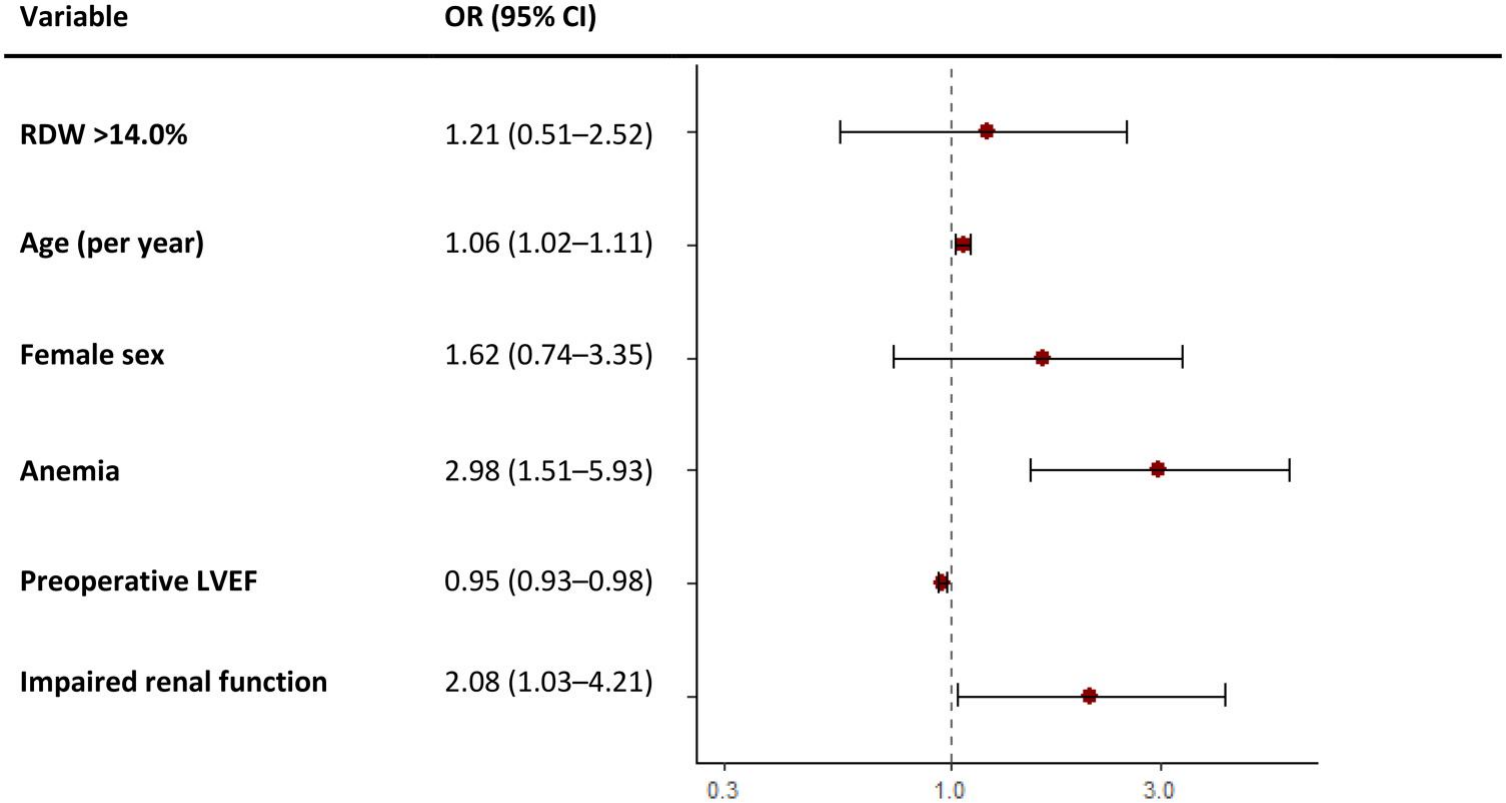
Forest Plot for Adjusted 30-Day Mortality. Abbreviations: LVEF = left ventricular ejection fraction; OR = odds ratio; RDW = red cell distribution width

Thirty-day mortality was 2.0% (*n* = 39). Crude mortality was higher with elevated RDW (4.1% vs 1.5%, *P* = .002) but lost significance after adjustment (OR 1.21, 95% CI 0.55-2.52), as shown in **Figure 4**. The mortality model had an AUC of 0.829. **Table S7** shows the results of uni- and multivariable logistic regression analyses of 30-day mortality.

### Other perioperative morbidity

Results for additional complications are shown in **Table 3**. Elevated RDW predicted increased use of platelet and plasma transfusion, prolonged ICU and hospital stay, and higher odds of major complications (**Table 3**). No associations were found with minor complications or postoperative AKI after adjustment. **Table S8** presents the distribution of complications by dichotomized RDW.

**Table 3.** Unadjusted and Adjusted Analysis of Perioperative Complications by Preoperative RDW

	Continuous RDW (per 1% increase)		Dichotomized RDW (>14.0% vs ≤14.0%)	
	Unadjusted OR (95% CI)	Adjusted^a^ OR (95% CI)	Unadjusted OR (95% CI)	Adjusted^a^ OR (95% CI)
Platelet transfusions ≥2 units	1.37 (1.23-1.52)	1.26 (1.10-1.43)	2.79 (2.00-3.87)	2.22 (1.51-3.25)
Plasma transfusions ≥2 units	1.23 (1.14-1.33)	1.29 (1.18-1.43)	1.79 (1.41-2.27)	1.81 (1.37-2.40)
Prolonged ICU LOS^b^	1.34 (1.23-1.46)	1.26 (1.13-1.40)	2.32 (1.80-2.99)	1.81 (1.33-2.47)
Prolonged total LOS^b^	1.13 (1.04-1.22)	1.12 (1.01-1.24)	1.64 (1.27-2.10)	1.44 (1.06-1.94)
Postoperative AKI^c^	1.18 (0.95-1.42)	1.08 (0.84-1.34)	1.61 (0.79-3.10)	1.03 (0.44-2.30)
Minor complications	1.09 (1.02-1.17)	1.03 (0.95-1.12)	1.37 (1.10-1.72)	1.14 (0.89-1.46)
Major complications	1.14 (1.03-1.25)	1.17 (1.05-1.30)	1.76 (1.31-2.35)	1.74 (1.23-2.45)

a Adjusted for preoperative aspirin, clopidogrel, heparin, and warfarin within 5 days; age, sex, BMI, year of surgery, preoperative anaemia, preoperative LVEF, preoperative eGFR, non-elective procedures, cross-clamp time, diabetes mellitus, COPD, and EuroSCORE II.

b Defined as LOS ≥75th percentile of the study cohort.

c Defined according to KDIGO criteria.

Abbreviations: AKI = acute kidney injury; BMI = body mass index; COPD = chronic obstructive pulmonary disease; eGFR = estimated glomerular filtration rate; EuroSCORE = European System for Cardiac Operative Risk Evaluation; ICU = intensive care unit; KDIGO = Kidney Disease Improving Global Outcomes; LOS = length of stay; LVEF = left ventricular ejection fraction; OR = odds ratio; RDW = red cell distribution width.

## DISCUSSION

In this nationwide cohort, elevated preoperative RDW was independently associated with greater bleeding risk after CABG, including higher transfusion needs, re-exploration, and chest tube output. Elevated RDW was also linked to greater perioperative morbidity, though not to 30-day mortality.

### Comparison with existing literature

Patients with high RDW typically share known bleeding risk factors—advanced age, anaemia, renal dysfunction, and urgent surgery^4–7^^,^^26^—which was also seen here. While RDW has been studied extensively as a predictor of mortality in cardiac surgery, its role in bleeding has been less clear. Prior work has reported associations with bleeding after PCI,^13^ gastrointestinal bleeding after CABG,^14^ and transfusion or re-exploration in small, open-heart surgical cohorts,^14^^,^^27^^,^^28^ whereas Warwick et al. found no such associations.^15^ Most other investigations have focused on mortality, with mixed results.^3^^,^^15^^,^^27^

Our findings extend this literature by showing a consistent association between elevated RDW and three separate bleeding outcomes in a large, national cohort—the first study, to our knowledge, to demonstrate associations across multiple detailed bleeding end-points. These findings extend RDW’s relevance to bleeding and align with reports linking it to impaired renal function,^26^ longer hospital stays,^26^ and multiorgan failure following cardiac surgery.^28^ Associations persisted in sensitivity analyses and across surgical eras; OPCAB analyses were underpowered but directionally similar. No significant RDW-surgical technique interactions were observed. Female sex and OPCAB status showed variable associations, likely reflecting collinearity with anaemia, urgency, and event distributions.

Mortality was not independently linked to RDW, likely due to the low mortality rate (2%), wide confidence intervals, and the dominant influence of age, comorbidities, and EuroSCORE II. RDW may therefore be more informative for morbidity and bleeding risk than for short-term mortality in contemporary CABG populations.

### Mechanism of increased bleeding

Red cell distribution width reflects anisocytosis, a hallmark of many anaemia subtypes. Although RDW correlated with lower haemoglobin and higher anaemia prevalence, its discriminative ability for anaemia was poor, suggesting RDW captures additional physiological processes beyond impaired erythropoiesis. Elevated RDW has been associated with adverse outcomes even in the absence of anaemia and may reflect underlying systemic stress, for example inflammation, nutritional deficiencies, and oxidative stress, which impair hemostasis and recovery capacity.^9^^,^^29^ The increased bleeding and complication rates seen with elevated RDW may reflect greater comorbidities (eg, older age, renal impairment, COPD) and overall physiological burden. Thus, RDW likely reflects underlying physiological stress and comorbidity burden rather than directly causing adverse outcomes, supporting its potential value as a complementary preoperative biomarker.

### Clinical implications

Although not incorporated into risk-stratification models such as EuroSCORE II,^19^ our findings suggest that RDW could have potential value in this context and might complement established predictors in future models. While our analysis was not designed to propose a new risk score, these results support evaluating RDW in future preoperative models.

### Strengths and limitations

Strengths of our study include its nationwide scope, comprehensive assessment of outcomes, and precise quantification of bleeding by transfusion and chest tube output. All CABG procedures were performed at a single institution, with cases being evenly distributed among surgeons, thus minimizing allocation bias. Importantly, all surgeons participate in the postoperative management of all patients, including transfusion decisions, rather than being limited to their own cases.

Limitations include the retrospective design that carries inherent risks of residual confounding. Inflammatory and nutritional markers (iron, vitamin B12, folate—which may influence RDW^30^) as well as platelet function testing, were unavailable. RDW data ended in 2019, and postoperative RDW values (eg, 3-6 months) were not captured, and we were therefore unable to assess the prognostic value of postoperative RDW evolution. Ticagrelor was not marketed in Iceland until 2017 and prasugrel in 2018, both towards the very end of our study period, and their uptake was minimal. Practice changes over 17 years, including lower transfusion thresholds adoption of plasma/platelet guidelines in 2010, may have influenced event rates, though associations persisted after adjusting for calendar year. The overall transfusion rate of 58% was higher than some contemporary series, reflecting both practice evolution and inclusion of urgent and emergent cases.

### Future directions

Further research should explore the biological links between RDW and perioperative outcomes, including its relationship with inflammatory markers and endothelial function. Future research should examine biological mechanisms linking RDW to outcomes, test its role in risk models, assess whether treating anaemia modifies risk, and validate findings in contemporary cohorts with standardized transfusion thresholds, longitudinal RDW data, and modern antiplatelet use.

## CONCLUSIONS

Elevated RDW is independently associated with increased bleeding complications after CABG and is thus a potential biomarker for preoperative risk stratification, giving opportunities to identify those at greater risk for bleeding and optimize perioperative management accordingly. Future studies that elucidate the biological mechanisms that underlie the association between RDW and adverse surgical outcomes are warranted.

## Supplementary Material

ivaf299_Supplementary_Data

## Data Availability

The data underlying this article cannot be shared publicly due to reasons of sensitivity. The data will be shared on reasonable request to the corresponding author.
