# Supplementary material for: Red Cell Distribution Width is Associated with Bleeding Complications after Coronary Artery Bypass Grafting
Source: Interdiscip Cardiovasc Thorac Surg. 2025 Dec 22;41(1):ivaf299. doi: 10.1093/icvts/ivaf299 (PMC12774467; doi:10.1093/icvts/ivaf299)
Supplement: ivaf299_Supplementary_Data [file ivaf299_supplementary_data.docx]

## Supplementary data

**Supplementary Table 1. The numbers (%) of missing data.**

| **Variable** | **N (%) with missing data** |
| --- | --- |
| **Red cell distribution width** | 0 (0) |
| **Sex** | 0 (0) |
| **Age** | 0 (0) |
| **Body mass index** | 0 (0) |
| **Left ventricular ejection fraction** | 57 (3.0) |
| **New York Heart Association score** | 0 (0) |
| **Canadian Cardiovascular Society score** | 0 (0) |
| **EuroSCORE II** | 0 (0) |
| **Three-vessel disease and/or left main stenosis** | 0 (0) |
| **Previous myocardial infarction** | 0 (0) |
| **Smoking status** | 39 (2.0) |
| **Chronic obstructive pulmonary disease** | 10 (0.4) |
| **Cardiac valve disease** | 1 (0.05) |
| **Hypertension** | 5 (0.2) |
| **Diabetes mellitus** | 12 (0.6) |
| **Preoperative glomerular filtration rate** | 0 (0) |
| **Preoperative hemoglobin** | 114 (5.9) |
| **Preoperative use of aspirin** | 90 (4.7) |
| **Preoperative use of clopidogrel** | 90 (4.7) |
| **Preoperative use of warfarin, heparin, low-molecular weight heparin** | 89 (4.6) |
| **Operative status (elective, urgent, emergent)** | 0 (0) |
| **Number of distal anastomoses** | 0 (0) |
| **Mammary artery used** | 0 (0) |
| **Cardiopulmonary bypass time** | 18 (1.1) |
| **Cross-clamp time** | 15 (0.8) |
| **Transfusion of RBC units** | 0 (0) |
| **Re-exploration for bleeding** | 0 (0) |
| **24-h postoperative chest tube output volume** | 0 (0) |
| **Length of stay, intensive care unit** | 0 (0) |
| **Length of stay, total** | 5 (0.3) |
| **Minor complications** |  |
| **Wound infection (superficial sternal or foot)** | 0 (0) |
| **Postoperative atrial fibrillation** | 0 (0) |
| **Pneumonia** | 0 (0) |
| **Urinary tract infection** | 0 (0) |
| **Major complications** |  |
| **Perioperative myocardial infarction** | 0 (0) |
| **Postoperative acute kidney injury requiring dialysis** | 0 (0) |
| **Deep sternal wound infection** | 0 (0) |
| **Stroke** | 0 (0) |
| **Sternal dehiscence** | 0 (0) |
| **Multiorgan failure** | 0 (0) |
| **30-day mortality** | 0 (0) |

Abbreviations: EuroSCORE, European System for Cardiac Operative Risk Evaluation.

**Supplementary Table 2. Incidences of each bleeding outcome according to preoperative RDW (normal vs. elevated).**

| **RDW groups** | **Normal RDW** | **Elevated RDW** | ***p*-value** |
| --- | --- | --- | --- |
| **Transfusion, n (%)** |  |  |  |
| Overall | 851 (55.2) | 269 (69.5) | **<0.001** |
| >4 RBC units | 191 (12.4) | 85 (22.0) | **<0.001** |
| **Re-exploration rate, n (%)** | 59 (3.8) | 34 (8.8) | **<0.001** |
| **Chest tube output >1000 mL/24h, n (%)** | 391 (25.4) | 98 (25.3) | 0.999 |

Abbreviations: RDW, red cell distribution width; RBC, red blood cell.

**Supplementary Table 3. Association between elevated RDW (>14.0%) and adjusted bleeding outcomes stratified by calendar period, 2003-2010 (n = 1141) vs. 2011-2019 (n = 815).**

| **Outcome** | **Time period** | **Continuous RDW (per 1% ↑)** | | **Dichotomized RDW (>14.0% vs. ≤14.0%)** | |
| --- | --- | --- | --- | --- | --- |
|  |  | **OR (95% CI)** | ***p*-value** | **OR (95% CI)** | ***p*-value** |
| **Transfusion >4 RBC units** | 2003-2010 | **1.14 (0.99–1.31)** | **0.058** | 1.24 (0.77–1.98) | 0.366 |
|  | 2011-2019 | **1.28 (1.03–1.58)** | **0.022** | **2.10 (1.22–3.61)** | **0.008** |
| **Re-exploration for bleeding** | 2003-2010 | **1.28 (1.02–1.58)** | **0.023** | **2.33 (1.14–4.62)** | **0.017** |
|  | 2011-2019 | 1.11 (0.81–1.45) | 0.498 | 1.64 (0.76–3.47) | 0.197 |
| **Chest tube output >1000 mL/24h** | 2003-2010 | 1.09 (0.96–1.23) | 0.200 | 1.16 (0.74–1.78) | 0.511 |
|  | 2011-2019 | 1.13 (0.94–1.34) | 0.178 | 1.31 (0.86–1.98) | 0.208 |

**Supplementary Table 4. Association between elevated RDW (dichotomized) and bleeding outcomes in on-pump (CPB) cases only (n = 1593).**

| **Outcome** | **Events, n (%)** | **Continuous RDW (per 1% ↑)** | | **Dichotomized RDW (>14.0% vs. ≤14.0%)** | |
| --- | --- | --- | --- | --- | --- |
|  |  | **OR (95% CI)** | ***p*-value** | **OR (95% CI)** | ***p*-value** |
| **Transfusion >4 RBC units** | 236 (14.8) | 1.28 (1.13–1.44) | <0.001 | 1.93 (1.35–2.77) | <0.001 |
| **Re-exploration for bleeding** | 86 (5.4) | 1.32 (1.12–1.55) | <0.001 | 2.67 (1.59–4.46) | <0.001 |
| **Chest tube output >1000 mL/24h** | 341 (21.4) | 1.19 (1.07–1.33) | 0.001 | 1.45 (1.06–1.98) | 0.020 |

Adjusted for age, sex, BMI, LVEF category, impaired renal function, anemia, diabetes, COPD, aspirin ≤5d, clopidogrel ≤5d, heparin ≤5d, warfarin ≤5d, operative urgency, CPB time, EuroSCORE II, and calendar year.

**Supplementary Table 5. Association between elevated RDW and bleeding outcomes in OPCAB cases only (n = 336).**

| **Outcome** | **Events, n (%)** | **Continuous RDW (per 1% ↑)** | |
| --- | --- | --- | --- |
|  |  | **OR (95% CI)** | ***p*-value** |
| **Transfusion >4 RBC units** | 40 (11.9) | 1.08 (0.77–1.48) | 0.621 |
| **Re-exploration for bleeding*** | 7 (2.1) | 1.10 (0.56–1.67) | 0.744 |
| **Chest tube output >1000 mL/24h** | 148 (44.0) | 0.92 (0.72–1.16) | 0.495 |

*Univariable analysis due to low number of events.

Otherwise adjusted for age, sex, BMI, LVEF category, impaired renal function, anemia, diabetes, COPD, aspirin ≤5d, clopidogrel ≤5d, heparin ≤5d, warfarin ≤5d, operative urgency, EuroSCORE II, and calendar year.

**Supplementary Table 6. Sensitivity analysis including preoperative platelet count (2005-2019, n = 1666). Multivariable logistic regression models of the association between RDW and bleeding outcomes, additionally adjusted for preoperative platelet count.**

|  | **Continuous RDW (per 1% increase)** | | **Dichotomized RDW (>14.0% vs. ≤14.0%)** | |
| --- | --- | --- | --- | --- |
|  | **Adjusted^a^ OR (95% CI)** | ***p*-value** | **Adjusted^a^ OR (95% CI)** | ***p*-value** |
| **Transfusion >4 units** | 1.27 (1.12–1.43) | <0.001 | 1.75 (1.22–2.49) | 0.002 |
| **Re-exploration** | 2.23 (1.13–3.71) | 0.016 | 2.23 (1.32–3.71) | 0.002 |
| **Chest tube output >1000 mL/24h** | 1.15 (1.03–1.28) | 0.013 | 1.34 (0.98–1.82) | 0.060 |

^a^ Adjusted for preoperative use of aspirin, clopidogrel within 5 days, heparin and warfarin; age, sex, BMI, year of surgery, preoperative platelet count, preoperative anemia, preoperative LVEF, preoperative eGFR, non-elective procedures, CPB status, diabetes mellitus, COPD, and EuroSCORE II.

Abbreviations: RDW, red cell distribution with; OR, odds ratio; CI, confidence interval; BMI, body mass index; LVEF, left ventricular ejection fraction; eGFR, estimated glomerular filtration rate; COPD, chronic obstructive pulmonary disease; EuroSCORE, European System for Cardiac Operative Risk Evaluation.

**Supplementary** **Table 7. Univariable and multivariable logistic regression analysis of 30-day mortality.**

|  | **Univariable** | | **Multivariable^a^** | |
| --- | --- | --- | --- | --- |
|  | **OR (95% CI)** | ***p*-value** | **OR (95% CI)** | ***p*-value** |
| **RDW, per 1% increase** | **1.43 (1.20–1.68)** | **<0.001** | 1.15 (0.91–1.41) | 0.214 |
| **RDW >14.0% vs. ≤14.0%** | **2.85 (1.46–5.41)** | **0.002** | 1.20 (0.55–2.50) | 0.639 |
| **Age, per year** | **1.09 (1.05–1.14)** | **<0.001** | **1.05 (1.01–1.10)** | **0.024** |
| **Female sex** | 1.89 (0.89–3.73) | 0.077 | 1.12 (0.49–2.37) | 0.770 |
| **Impaired renal function** | **4.71 (2.48–8.99)** | **<0.001** | 1.65 (0.75–3.53) | 0.201 |
| **EuroSCORE II, per %** | **1.17 (1.13–1.21)** | **<0.001** | **1.14 (1.09–1.19)** | **<0.001** |

^a^ Adjusted for age, sex, impaired renal function, and EuroSCORE II.

Abbreviations: OR, odds ratio; CI, confidence interval; EuroSCORE, European System for Cardiac Operative Risk Evaluation.

**Supplementary Table 8. Incidence of perioperative complications according to preoperative RDW.**

|  | **Normal RDW ≤14.0% (n = 1542)** | **Elevated RDW >14.0% (n = 387)** | ***p*-value** |
| --- | --- | --- | --- |
| Prolonged ICU LOS | 255 (16.5) | 122 (31.5) | **<0.001** |
| Prolonged total LOS | 315 (20.5) | 115 (29.7) | **<0.001** |
| Postoperative AKI^a^ | 82 (5.3) | 32 (8.3) | **0.037** |
| Minor complications, total | 727 (47.1) | 213 (55.0) | **0.006** |
| Superficial wound infection (sternal or foot) | 153 (9.9) | 44 (11.4) | 0.446 |
| Postoperative atrial fibrillation | 575 (37.3) | 166 (42.9) | **0.049** |
| Pneumonia | 90 (5.8) | 37 (9.6) | **0.011** |
| Urinary tract infection | 39 (2.5) | 28 (7.2) | **<0.001** |
| Major complications, total | 193 (12.5) | 78 (20.2) | **<0.001** |
| Perioperative MI | 66 (4.3) | 8 (2.1) | 0.062 |
| Postoperative AKI requiring dialysis | 14 (1.0) | 9 (2.3) | **0.041** |
| Deep sternal wound infection | 12 (0.8) | 7 (1.8) | 0.122 |
| Stroke | 17 (1.1) | 5 (1.3) | 0.963 |
| Sternal dehiscence | 18 (1.2) | 5 (1.3) | 0.795 |
| Multi-organ failure | 31 (2.0) | 23 (5.9) | **<0.001** |

Mean ± standard deviation, median [IQR] or number (%).

^a^ Defined according to KDIGO criteria.

Abbreviations: RDW, red cell distribution with; ICU, intensive care unit; LOS, length of stay; AKI, acute kidney injury; MI, myocardial infarction; IQR, interquartile range; KDIGO, Kidney Disease Improving Global Outcomes.


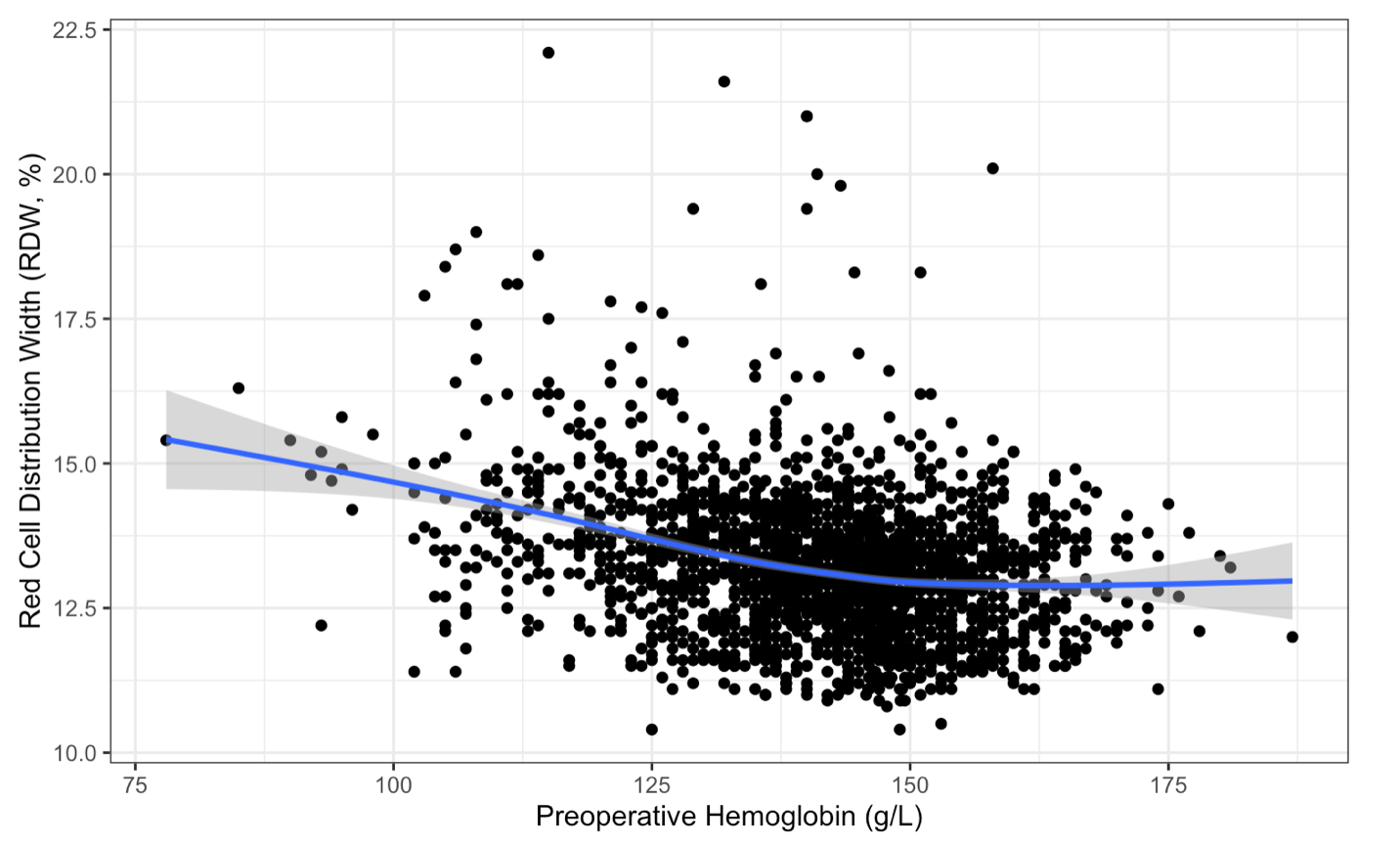


**Supplementary Figure 1.** Scatterplot of preoperative hemoglobin (g/L) and RDW (%) with a fitted spline curve. RDW shows an inverse linear association with hemoglobin for values <150 g/L, with a plateau at higher hemoglobin levels.


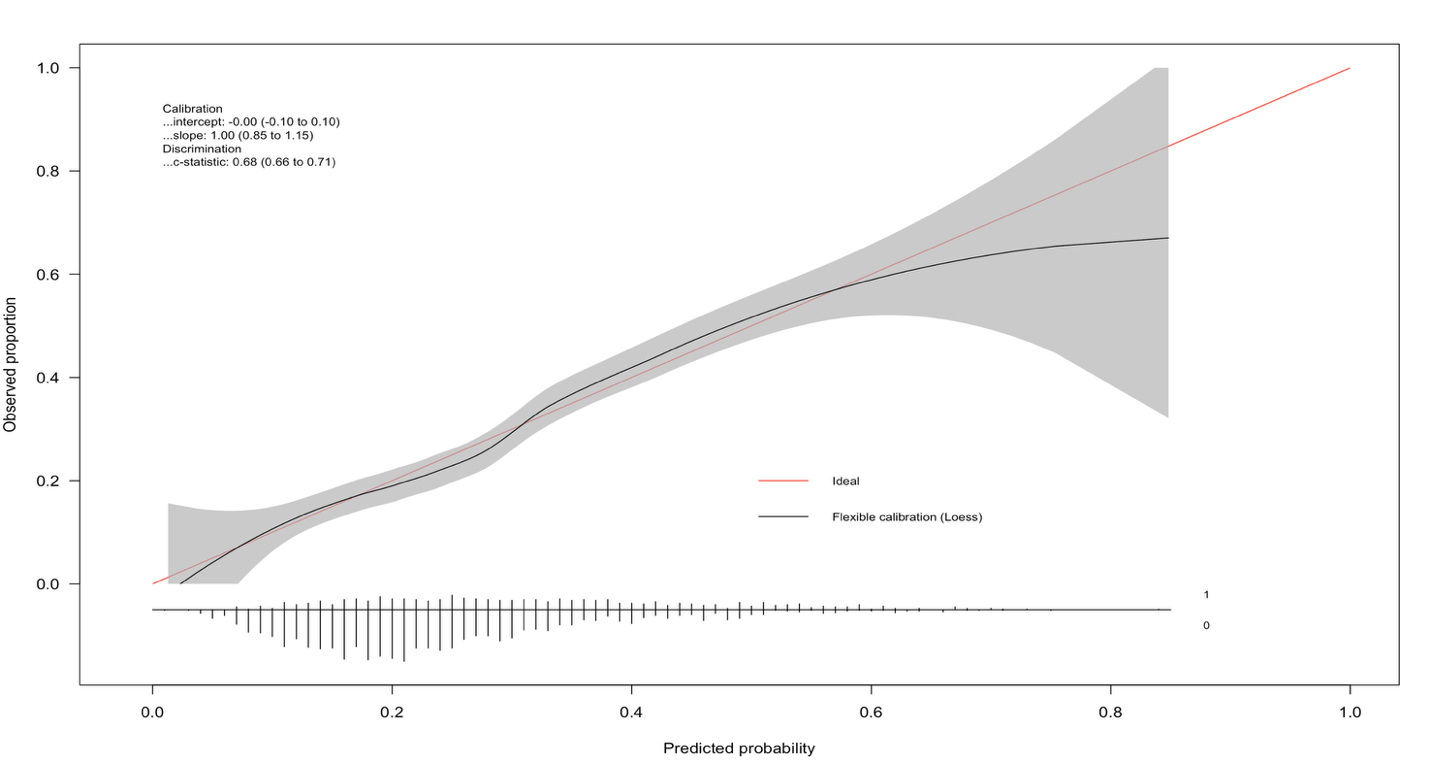


**Supplementary Figure 2.** Calibration curve for the chest tube bleeding model.


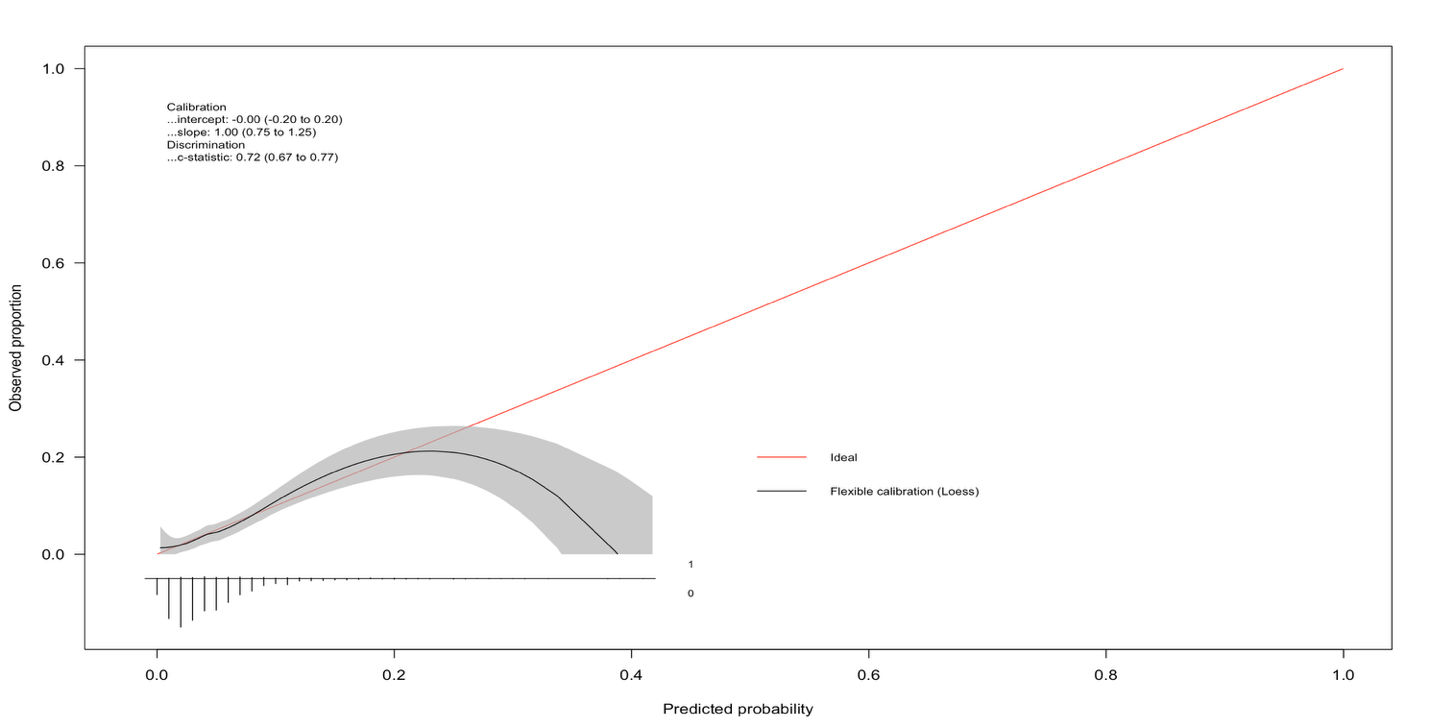


**Supplementary Figure 3.** Calibration curve for the re-exploration model.


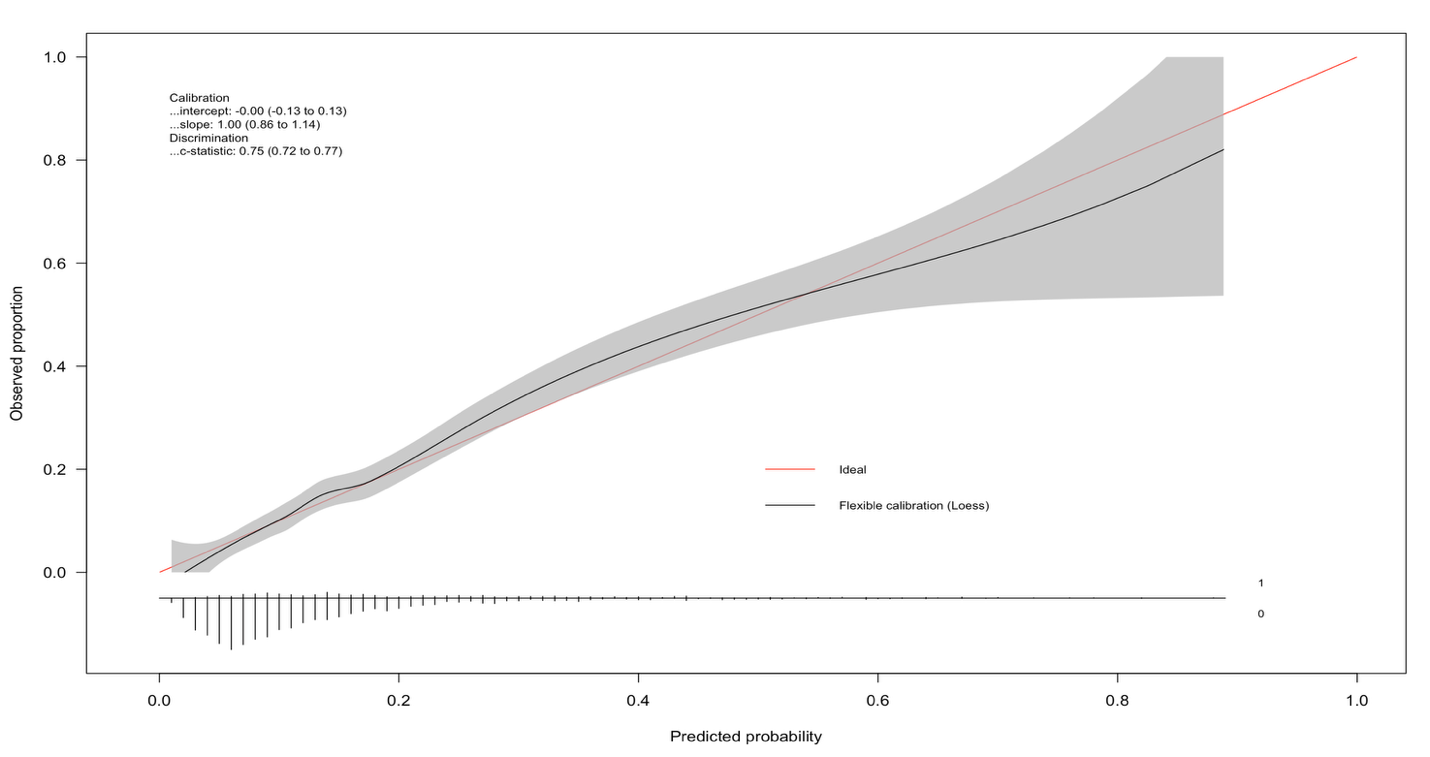


**Supplementary Figure 4.** Calibration curve for the tranfusion model.
